# Application of population pharmacokinetics to cladribine

**DOI:** 10.1186/1471-2210-5-4

**Published:** 2005-03-09

**Authors:** Synnöve Lindemalm, Radojka M Savic, Mats O Karlsson, Gunnar Juliusson, Jan Liliemark, Freidoun Albertioni

**Affiliations:** 1 Department of Oncology-Pathology, Cancer centre Karolinska, Karolinska Institute, Stockholm, Sweden; 2 Division of Pharmacokinetics and Drug Therapy, Department of Pharmaceutical Biosciences, Uppsala University, Uppsala, Sweden; 3 Department of Haematology, Linköping University Hospital, Linköping, Sweden; 4 Department of Clinical Pharmacology, University Hospital, Linköping, Sweden

**Keywords:** Leukemia, Cladribine, Pharmacokinetic, NONMEM, Clearance

## Abstract

**Background:**

The nucleoside analog cladribine is used for the treatment of a variety of indolent B- and T-cell lymphoid malignancies. The primary aim of the study was to evaluate the population distribution of pharmacokinetic parameters in patients undergoing treatment with cladribine and to detect the influence of different covariates on the pharmacokinetic parameters.

**Methods:**

This pharmacokinetic study presents the results of a retrospective population pharmacokinetic analysis based on pooled data from 161 patients, who were given cladribine in different administration routes in various dosing regimens. The plasma concentrations of cladribine were determined by reversed-phase high-performance liquid chromatography using a solid phase extraction with a limit of quantitation of 1 nM using 1 mL of plasma.

**Results:**

A three compartment structural model best described the disposition of cladribine. Clearance was found to be 39.3 L/hour, with a large interindividual variability. The half-life for the terminal phase was 16 hours. Bioavailability was 100% and 35% for subcutaneous and oral administration, respectively, with low interindividual variability. None of the investigated covariates were found to be correlated with the pharmacokinetic parameters.

**Conclusion:**

As interindividual variability in apparent clearance after oral administration was not significantly higher compared to that following infusion, cladribine could be administered orally instead of intravenously if compensated for its lower bioavailability. Individualized dosing on basis of body surface area or weight does not represent an improvement in this study as compared to administering a fixed dose to all patients.

## Background

Cladribine [Leustatin^®^] is a purine analogue that entered clinical testing fifteen years ago, with major activity in the treatment of B- and T-cell lymphoid malignancies. Cladribine has an outstanding therapeutic activity against hairy cell leukemia, a disease in which the drug induces long-lasting complete remissions in the vast majority of patients treated. The activity of cladribine has also been demonstrated in chronic lymphocytic leukaemia (CLL) non-Hodgkin's lymphoma, cutaneous T-cell lymphoma and myeloid leukemia. Cladribine and the other newer purine analogues are unique, when compared to traditional antimetabolites, in that they are equally cytotoxic to both dividing and resting cells [1]. Cladribine is usually administered at 0.09 mg/kg daily as a continuous intravenous infusion over 7 days. However, pharmacokinetic studies supporting the use of intermittent intravenous (iv) infusions have shown a long terminal half-life of cladribine after a 2-hour infusion with the same anti tumour activity seen with continuous iv infusion [2]. The pharmacokinetic profile of oral administration of cladribine resembles that of a 2-hour iv infusion, with a bioavailability of 37–51% [3,4]. Subcutaneous administration gives a high peak concentration of short duration with an area under the curve (AUC) identical to that of the iv infusion and a bioavailability of 100%.

The primary aim of the study was to evaluate the population distribution of pharmacokinetic parameters in patients undergoing treatment with cladribine and to detect the influence of different covariates on the pharmacokinetic parameters. This analysis was performed in order to evaluate the plasma concentration-time profiles in relation to previously presented pharmacokinetic data and to elucidate the possibilities to create a better tailoring of cladribine dosing.

## Results

**Figure 1:**
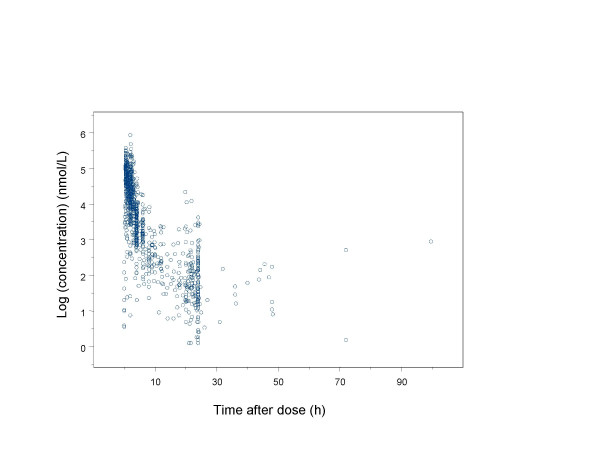
Observed plasma concentration versus time profile for cladribine after a 2- hour iv infusion, oral and subcutaneous administration once daily. Concentrations are given on a logarithmic scale.

**Figure 2:**
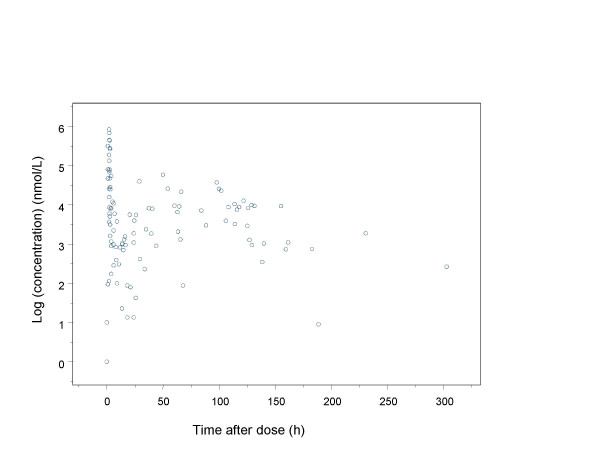
Observed plasma concentrations after repeated intravenous administration (steady -state observations). Concentrations are given on a logarithmic scale.

The population pharmacokinetic analysis of cladribine was based on 1102 plasma concentrations obtained from 161 individuals. The observed plasma concentrations of cladribine versus time are presented in Figure 1 and 2. The initial runs carried out were aimed at finding a base model (pharmacokinetic and statistical submodels). A three compartment structural model best described the time course of plasma concentrations of cladribine for all patients and was therefore chosen for the present analysis.

The final disposition model is described as follows: a three compartment model with interindividual variability on clearance (*CL*), central and peripheral volumes of distribution (*V1*, *V2*, *V3*), intercompartmental clearances (*Q2, Q3*), and with a proportional residual error model for residual variability.

**Figure 3:**
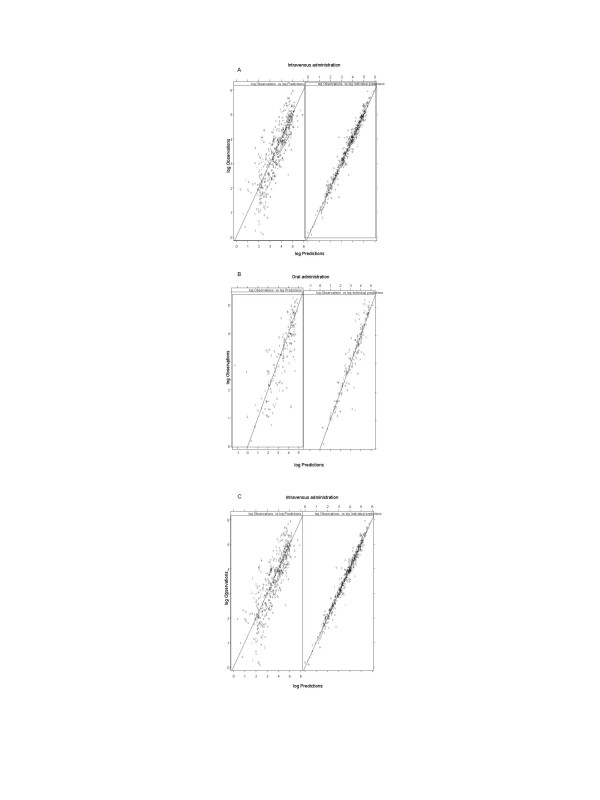
Model predicted versus observed cladribine concentrations after intravenous infusion (A), oral (B) and subcutaneous (C) administration. In the left panel predictions are based on the parameters of the typical individual whereas in the right panel predictions are based on individual parameter estimates. ID numbers has been used as plotting symbols and all observations for an individual are connected by a broken line. Concentrations are given on a logarithmic scale to facilitate model inspection.

The final population parameter estimates based on the model are given in Table 3. The clearance in the typical patient was calculated to be 39.3 L/h, with relative standard error (RSE) of 4.9 % while the interindividual variability, as expressed by the coefficient of variation, was 54%. Diagnostic plots of the observed and population model predicted concentrations after iv infusion, oral and subcutaneous administrations are shown in Figure 3A–C.

**Table 3:** Population pharmacokinetic parameter estimates for the typical individual after administration of cladribine as; an infusion, orally or subcutaneously. The relative standards errors are given in parentheses. The estimates of intersubject variability are given as coefficients of variation (%).

**Parameter**	**Population average**		**Interindividual variability**	
	Estimate	(RSE%)^1^	Estimate %	(RSE%)
Clearance (L/h)	39.3	(4.9)	54	(17)
V1 (L)	71.7	(13)	34	(62)
Q2 (L/h)	51.1	(6.8)	61	(17)
V2 (L)	475	(1.8)	70	(31)
Q3 (L/h)	105	(21)	61	(17)
V3 (L)	73.6	(13)	61	(17)
Oral Ka (h^-1^)	1.31	(14)	75	(50)
Oral F	0.353	(7.9)	4.1	(63)
Subcutaneous Ka (h^-1^)	2.48	(9,6)	N.E.	
				

**Parameter**	**Infusion (RSE %)**	**Oral (RSE %)**	**Subcutaneous (RSE %)**	**IIV^3 ^(RSE %)**

Residual error^2^				
Proportional	0.191 (8.2)	0.232(9.8)	0.162 (8.8)	24 (28)

^1 ^Rellative standard error. given as %.

^2 ^Residual error presented as population average of estimate and relative standard error in parentheses.

^3 ^Interindividual variability in residual variability expressed as coefficient of variation

Abbreviations: CL, clearance from central compartment; *V1*, *V2 *and *V3*, central and peripheral volumes of distribution, Q2 and Q3, intercompartmental clearances; Ka, absorption rate constant (hour^-1^); F, bioavailability of drug; N.E., not estimated

The oral data were best described with a first order absorption without lag time. The bioavailability was found to be 0.353 with RSE 7.9% and the interindividual variability was estimated to be 4 % (expressed as coefficients of variation). The estimated value for the absorption rate constant was 1.31 hour^-1 ^with RSE 14%. As bioavailability and interindividual variability in F were of particular interest, some additional investigation was performed. First, the 95% confidence interval for interindividual variability in F was obtained using likelihood profiling. This resulted in a 95 % confidence interval ranging from 0% to 10%. Second, in order to get a broader insight into the first pass metabolism, bioavailability and variability in CL following different administration routes, F was omitted from the model and instead apparent CL and its interindividual variability was estimated separately for each route of administration (other disposition parameters were still estimated jointly). The estimated CL for the intravenous route was 39.7 L/h (RSE 8%) while the estimates of the oral CL (or rather CL/F) was 105 L/h (RSE 13%). The estimated interindividual variability in CL for the intravenous and oral routes were 59% (RSE 22%) and 55 (50%) respectively. Third, a model allowing correlation between F and CL was tested, but it did not result in any improvements and variability in F was still not appreciable.

For the subcutaneous data the bioavailability was calculated to be close to 1 and therefore was fixed to this value, as estimation of this parameter did not result with a significant model improvement. The corresponding result for ka was 2.48 hour^-1 ^with RSE 9.6%.

The terminal half-life was relatively long, 16 hours. The half-life for the first distribution phase was calculated to be 0.2 hour and the corresponding half-life for the second distribution phase was 1.3 hours.

The highest and the lowest values for terminal half-life were 58 hours and 5 hours respectively. The corresponding accumulation index, the amount at steady state compared to the corresponding value after the first dose at the same time, was for the highest 3.05 and for the lowest 1.02.

None of the covariate relations, included in the generalised additive model, were found to be significant when included in the NONMEM model.

## Discussion

In this study we have evaluated pooled data from 161 patients at different centres, receiving different doses of cladribine, administered by three different routes, using different treatment schedules and leaving different numbers of blood samples at different times. To perform this evaluation we used nonlinear mixed effect modelling for the pharmacokinetic analysis of the data. A three compartment structural model best described the time course of plasma concentrations of cladribine, which is also in agreement with previously published individual pharmacokinetic modelling [4,5]. Cladribine, administered as an oral solution, was rapidly absorbed and the absorption was best described using a model with first order absorption without lagtime.

We calculated that the bioavailability after oral administration of cladribine was 35.3% and this value is slightly lower compared to the previous reports of 37–51% [3,4,6,7]. The estimate of interindividual variability (IIV) in oral bioavailability was surprisingly low, 4%. Only seven patients had data following both intravenous and oral administration and even if in theory, bioavailability and variability in bioavailability can be estimated from a parallel group study, the sparsity of crossover data might be one explanation for the negligible value of variability in bioavailability and a lower value for bioavailability.

Further, as all variability estimates are based on the nominal dose being exact, deviations from this would inflate variability in pharmacokinetic parameters. Therefore, another possible explanation is that the apparent lack of variability in F is due to that the variability between the nominal and actual dose is larger for intravenous administration than for oral administration. Variability between nominal and exact dose will arise in the manufacturing process, but may also occur due to deviations between nominal and actual volume infused and number of tablets administered.

Last, estimates of variability in bioavailability obtained by traditional non-compartmental methods are upwards biased as any error in determining the AUC following intravenous or oral administration will be translated into an individual bioavailability estimate differing from the true one. Thus, even a drug without variability in bioavailability will appear to have variability when this is estimated by classical two-stage methods. Nonlinear mixed effects methods are in general showing less bias in variability estimates than the classical two-stage methods [8]. Many factors contribute to the bioavailability of cladribine, but the low variability in bioavailability was confirmed with classic pharmacokinetics (data not shown), which is also in agreement with previous analyses [3,4]. Consequently, the variability in AUC after oral administration is of the same magnitude as after intravenous administration, as the variability in CL is more pronounced than that for the bioavailability Table 3.

To reach the general circulation, a drug given orally must pass through the liver via the portal system. A shorter onset and a more intense response may occur when giving a compound orally, rather than as a 2-hour intravenous infusion, if the compound is rapidly absorbed and undergoes extensive first-pass conversion to an active metabolite more potent than the parent. The metabolite of cladribine, 2-chloroadenine, has 8 times lower cytotoxic effect than cladribine, while five times more metabolite is formed after an oral administration compared with after an iv infusion [9].

After subcutaneous administration the bioavailability was found to be 100% with a low interindividual variability less than 1%. These results are in agreement with the previous analysis [3,7].

For the typical patient, 65 % of the elimination is associated with the terminal slope, 11% with the first and 24% with the second slope. Although distribution kinetics cannot be ignored, the majority of cladribine elimination is clearly associated with events defined by the terminal phase, which has a mean half-life of 16 hours.

It is standard practice in oncology to individualise chemotherapy dosing, and to dose according to BSA or weight, with the aim of reducing the interpatient variability of drug effect and toxicity. However, neither weight nor BSA explains more than a minor part of the variability seen. In this study, the modelling indicates that dosing according to body weight or BSA does not represent any significant improvement as compared to administration a fixed dose to all patients. For a convincing evaluation of dosing cladribine, more patients need to be studied, but the lack of reduction in interindividual variability when entering relationships with body size make it unlikely that body size can explain more than a small portion of the interindividual variability. Likewise, it has been shown that BSA fails to standardize the marked interpatient variation in pharmacokinetic variables for most cytotoxic drugs [10]. There is no simple satisfactory method for calculating drug dose, and other non-BSA-based dose calculation methods have been proposed and discussed [10-14]. Patients with a dose corrected for BSA or weight, but with a low clearance, tend to have an excessive accumulation of the drug and a high risk of toxicity. The amount at any time within the dosing interval at plateau was maximum 3.05 times and minimum 1.02 times the values at the corresponding times after a single dose. It seems therefore valuable to adjust the dose according to pharmacodynamic events such as observed toxicity, which is one parameter used in non-BSA-based dose calculation methods to individualise treatment [10]. This is possible when patients are treated with repeated courses, e.g. in low-grade lymphomas, but not in the treatment of hairy cell leukemia where only one course of treatment is given.

The reasons why people differ in their responsiveness to drugs are manifold. Age, weight, height, disease stage etc. are important because they are sources of variability that can be taken into account. None of the covariates included in this study was found to have a significant influence on the pharmacokinetics of cladribine. In this study no patient with severe reduction of liver- or kidney function was participating. Separate studies with such patient groups are warranted.

## Conclusion

As interindividual variability in apparent clearance after oral administration was not significantly higher compared to that following infusion, cladribine could be administered orally instead of intravenously if compensated for its lower bioavailability. Individualized dosing on basis of body surface area or weight does not represent an improvement in this study as compared to administering a fixed dose to all patients.

## Methods

### Patients

This population pharmacokinetic study is based on drug concentration data from previously published pharmacokinetic studies [2,3,5-7]. Patients from five centres in Sweden, Department of Oncology and Department of Hematology at Karolinska Hospital in Stockholm, Department of Medicine at Huddinge Hospital in Stockholm, Department of Hematology at Linköpings Hospital in Linköping and one centre in the United Kingdom, Taunton Hospital, Somerset, participated in these studies after giving their informed consent. The study period, from February 1990 to March 1996, included 215 patients and 227 courses. Fifty-three courses in 52 patients were excluded due to lack of information. Thus, 173 courses in 163 patients, (129 male, 34 female) with a mean age of 60 years (range 22–89) were initially included in population analysis. However, during the analyses, one subject was omitted as concentration time profile showed seven-fold increase after ending the infusion and one subject was excluded as an outlier in that the absorption was considerably slower than for other individuals, possibly indicating another site of administration. Two courses were excluded as drug was given in other administration route (rectal) than the ones that are of interest for the current analysis. Thus the final population analysis included 168 courses in 161 patients. More details regarding demographics and distribution of the different diagnoses among patients are presented in Table 1. The intravenous infusion results were based on 93 doses during 63 courses, the subcutaneous results on 83 doses during 83 courses and the oral results on 24 doses during 22 courses. For seven patients observed concentrations following both intravenous and oral administration was available. There was no difference in demographic data between patients included and not included in the analysis.

**Table 1:** Descriptive statistics and distribution of diagnoses for patients initially included in the population pharmacokinetic analysis.

	**Gender **m/f	**Age **(years)^2^	**Weight **(kg)^2^	**Height **(cm)^2^	**BSA**^1 ^(m^2^)^2^	
**Total**	129/34	60 (13) [22–89]	76 (14) [48–118]	174 (9) [152–198]	1.9 (0.2) [1.5–2.4]	
						
						
**Diagnosis**	**CLL**	**HCL**	**AML**	**NHL**	**CML**	**LCH**
**Total**	63	84	3	7	4	2

^1 ^body surface area

^2 ^data is presented with mean (SD)

Abbreviations: CLL, chronic lymphocytic leukemia; HCH, hairy cell leukemia; AML, acute myeloic leukemia; NHL, non-Hodgkin's lymphoma; CML, chronic lymphocytic leukaemia; LCH, Langerhan's cell histiocytosis.

#### Drug administration

The trials, on which this study was based, had previously been approved by the local Ethics Committee at the Karolinska Institute (Dnr 91:4, 91:190, 92:41, 93:62) and by the Swedish Medical Product Agency.

The dose for the *iv *administration (2-hour infusion) was 5 mg/m^2 ^or 0.12 mg/kg. The corresponding oral dose was 10 mg/m^2 ^or 0.24 mg/kg administered in saline after overnight fasting. No food was allowed until two hours after dosage. The subcutaneous dose, 5 mg/m^2 ^(2 mg/mL) was given in the adipose tissue in the abdominal wall as an injection. Six patients received a continuous iv infusion for four to seven days, with a dose between 4.0–5.6 mg/m^2^/24 hours. Three patients had a 2-hour iv infusion the 1st day and a continuous iv infusion the 2nd day with a dose of 4.8–5.7 mg/m^2^. Dosing and sampling history was collected including the dosing date and time, dose, and treatment period.

The covariate data collected were gender, age at treatment, body weight, body height, diagnosis and are summarized in Table 1. Also laboratory data was included in the analysis, creatinine clearance according to Cockcroft-Gault formula [15] presented kidney function, liver function tests and lymphocyte count. There was no difference in the laboratory data between the different routes of administration. The laboratory data for the mean patient presented with standard deviation and range are presented in Table 2.

**Table 2:** Distribution of laboratory data for patients included in the population pharmacokinetic analysis

	**Mean**	**SD**	**Range**	**Outliers**
Creatinine clearance (mL/min)	81	27	5–162	5^i^, 16^i^, 27^i^, 161^sc^, 162^i, o^
S-aspartate aminotransferase (μkat/L)	0.52	0.44	0.07–4.4	1.9^i^, 2.1^sc^, 2.2^i^, 4.4^i^
S-alanine amintransferase (μkat/L)	0.49	0.5	0.08–5.3	1.6^i^, 2.0^i^, 2.1^sc^, 5.3^i^
S-alkaline phosphatase (μkat/L)	1.3	6.7	1.0–64	12^i^, 12^i^, 14^sc^, 54^i^, 64^i^
S-bilirubin (μmol/L)	14	25	3.0–284	41^sc^, 88^i^, 284^i^

Outliers were defined as: Creatinine clearance <30->150 (mL/min), aspartate aminotransferase >1.5 (μkat/L), alanine amintransferase >1.5 (μkat/L), alkaline phosphatase >10 (μkat/L), bilirubin >30 (μmol/L)

Abbreviations:i, infusion; o, oral; sc, subcutaneous

The lymphocyte count was for the mean patient with CLL 124(123) [3.2-472] × 10^9^/L. CLL patient's response to treatment was collected and was distributed as 32% complete remission, 30% partial remission and 38% no remission in 73 patients. Staging according to Rai was 23% stage 1, 27% stage 2, 19% stage 3 and 30% stage 4 and staging according to Binet was 19% A, 33% B and 48% C. Response, staging according to Rai and Binet were included in the analysis of the 73 CLL patients. These data were collected at the beginning of the treatment.

#### Blood sampling

Venous samples (10 mL) were collected from an indwelling catheter into heparinized Venoject^® ^tubes. The blood was stored in ice water. The plasma was isolated within 6 hours after sampling and frozen immediately at -20°C. The average patient had 8 (range 1–27) samples taken up to 36 (range 3–303) hours.

#### Determination of cladribine in plasma

The plasma concentrations of cladribine were determined by reversed-phase high-performance liquid chromatography using a solid phase extraction [2,16]. The limit of quantitation was 1 nM for cladribine using 1 mL of plasma, when determined cladribine at 265 nm.

#### Population pharmacokinetic analysis

Nonlinear mixed effect modelling was applied for the pharmacokinetic analysis of the data using the software NONMEM[17]. The program Xpose [18] was used for data set checkout, exploration and visualisation, model diagnostics, candidate covariate identification and model comparison.

#### Structural pharmacokinetic model

The model building strategy involved a development of an integrated model that simultaneously fit the data from all administration routes. Thus, the estimates from this model of the population disposition parameters and interpatient variabilities are based on data from all patients receiving cladribine by all administration routes.

Two- and three compartment disposition models were evaluated for cladribine. A first-order absorption model, characterized by the absorption rate constant (ka) with estimated bioavailability (F) was used for extravascular administration. The presence of a lag-time was investigated for oral administration.

The analyses were made using the first order conditional estimation method with interaction in NONMEM.

#### Statistical model

The interpatient variability for the parameters was described using exponential models. Interpatient variability was included on clearance (CL), central volume of distribution (V1), peripheral volumes of distribution (V2 and V3), intercompartmental clearance (Q2 and Q3), absorption rate constant (ka) and bioavailability of drug (F). Covariances between interpatient variabilities in different pharmacokinetic parameters were investigated.

Residual variability, which represents the composite influence of assay variability, patient compliance and model misspecification, were described as a proportional component with separate estimates for the different routes of administration. The residual errors were assumed to be symmetrically distributed. Further, it was assumed that the residual error may not be constant across the individuals since data were pooled from different centres and collected over a long period of time. In order to handle such a variation in the residual variability, the individual contribution to the residual error was accounted for by including an interpatient variability in the residual error model [19].

#### Covariate model

The relationships between individual pharmacokinetic parameters and covariates were explored using the software Xpose and auxiliary program PsN [20]. The covariate model was built in a stepwise fashion within the population model, in which both linear and nonlinear relationships between the pharmacokinetic parameters and covariates were considered. The algorithm and assumptions have been described in detail elsewhere [21]. A generalised additive model was used to analyse the relationship between covariates and preliminary individual parameter estimates [22]. This procedure, based on the Akaike information criteria [23], will search for significant relationships between each of the parameters and the candidate covariates. These models guided the inclusion of covariates into the population model. The identified candidate covariates were evaluated in the base model by testing each covariate individually on each parameter and then by testing combinations of covariates on the parameters. The P level for inclusion of a covariate into the final population model was 0.01. This test was based on the objective function value produced by NONMEM, which is minus twice the Log Likelihood value. The difference in the objective function value between hierarchical models is approximately chi-squared distributed.

#### Model diagnostics and validation

Basic goodness of fit plots including population and individual predictions versus observed concentrations, as well as individual predictions versus individual weighted residuals, were checked for diagnostic purposes. The population predictions are based on the typical population parameters in the final population model, and the individual predictions on the empirical Bayes estimates of individual parameters.

#### Other calculations

The extent of accumulation following intravenous administration was obtained through model simulations and was based on AUC following the first and a steady state dosing interval.

## Authors' contributions

All authors participated in designing the study and/or writing the paper. Physician in charge of cladribine chemotherapy were JL and GJ. SL, RS and MOK were responsible for pharmacokinetic analyses. SL had the primary responsibility for the data collection. We thank Mats Strömberg for his skilful and reliable technical assistance and the staff at the Department of Oncology and Department of Haematology, Karolinska Hospital, for performing the blood sampling. We also like to thank Birgitta Pettersson for performing HPLC analysis.
